# Mapping auxin dynamics in the formation of lateral roots with a dual-DR5 reporter

**DOI:** 10.1093/plphys/kiaf447

**Published:** 2025-09-27

**Authors:** Blanca Jazmin Reyes-Hernández

**Affiliations:** Assistant Features Editor, Plant Physiology, American Society of Plant Biologists; Faculty of Science, Department of Plant and Environmental Sciences, Section for Plant Glycobiology, University of Copenhagen, Frederiksberg C 1871, Denmark

Roots grow downward and branch outward through the process of lateral root (LR) formation, building a root system that secures water and nutrients. In most flowering plants, LRs appear after germination along the primary root in a regularly spaced left–right pattern linked to gravity-induced waving (De Smet et al. 2007). Along the root, 3 basic zones extend from tip toward the shoot: (i) the meristematic zone, or root apical meristem, where cells divide; (ii) the elongation zone, where cells rapidly increase in length; and (iii) the differentiation zone, where mature cells acquire specific functions (Fig. 1) (Ivanov and Dubrovsky 2013).

**Figure 1.**
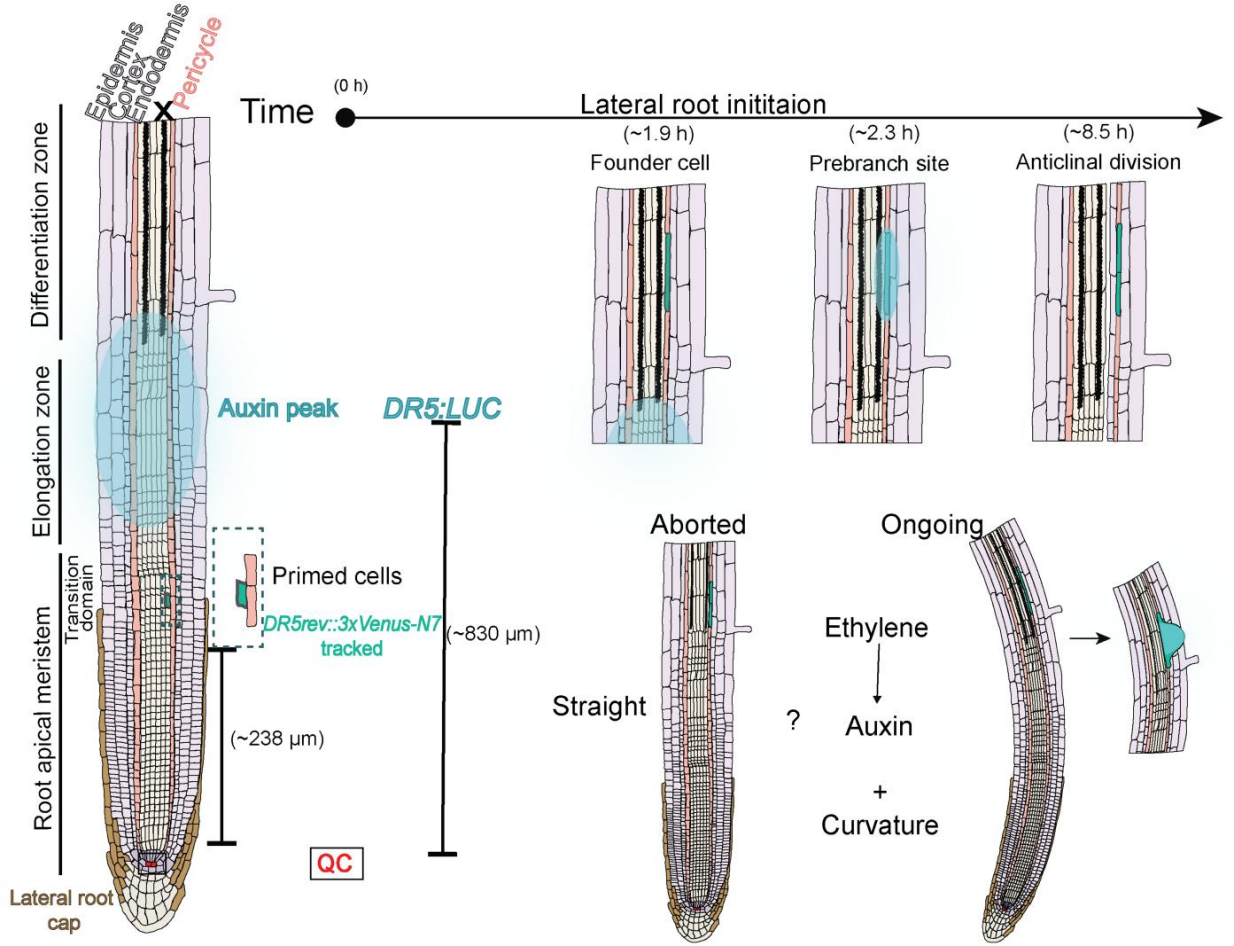
Description of spatio-temporal dynamics during lateral root organogenesis based on the dual-DR5 reporter from De Gernier et al. (2025). Cells leaving the apical meristem become primed for lateral root organogenesis within the transition domain, as revealed by the *DR5rev::3XVENUS-N7* signal. These primed cells migrate through the elongation zone before reaching the differentiation zone. At this stage, the auxin response, measured as *DR5::LUC* activity (auxin peak) and likely occurring in protoxylem pole (X) cells, increases and reaches a maximum. On average, priming occurred ∼238 *µ*m from the quiescent center (QC), whereas auxin peaks were detected further shootward, at ∼830 *µ*m. Founder cell specification, marked by pericycle fluorescence, appeared ∼1.9 h later. Prebranch sites became evident ∼2.25 h after the peak, and the first anticlinal division took place ∼8.5 h later. Iteration of this sequence over time is thought to underlie the regular spacing of lateral roots along the primary root. When comparing aborted and ongoing prebranch sites, aborted sites, mainly located in straight parts of the root, displayed weaker and delayed *DR5::LUC* increases, although the timing of founder cell specification was similar. The amplitude of the auxin peak and the ∼2.5 h interval between peaks did not differ significantly between the two types of sites.

In *Arabidopsis*, extensive studies have identified a sequence of developmental steps in the LR formation process: priming, founder-cell specification, LR initiation, LR primordium (LRP) morphogenesis, and LR emergence. After these steps, a new LR meristem becomes active and then the new LR grows. Each of these steps occurs progressively farther from the parental root elongation zone toward the differentiation zone (Torres-Martínez et al. 2022).

Priming occurs at the transition zone, which is near the boundary between the root apical meristem and the elongation zone (Fig. 1) (Ivanov and Dubrovsky 2013). Here, certain cells of the pericycle (outer layer of the vascular cylinder) adjacent to the protoxylem pole gain the potential (becoming “primed”) to later develop into founder cells (De Smet et al. 2007). During LR initiation, the founder cells begin to grow and divide. As development proceeds, the dividing cells form a dome-shaped LRP that pushes through the endodermis, cortex, and epidermis until it emerges from the parental root (Torres-Martínez et al. 2022).

Priming is closely linked to changes in auxin signaling, which regulates key developmental processes, including root development. Changes in auxin signaling and concentrations are often monitored using reporter genes controlled by the synthetic auxin-responsive promoter *DIRECT REPEAT5* (DR5; Ulmasov et al. 1997). During priming, auxin reporter activity first appears in the protoxylem (early xylem in the root vasculature) and then in nearby pericycle cells (De Smet et al. 2007). Subsequently, auxin-responsive transcription shows rhythmic increases and decreases within the elongation zone, hence the term “oscillation zone.” These rhythmic increase and decrease pulses repeat every few hours, so particular cells moving away from the root tip typically encounter 1 maximun (auxin peak) of the rising auxin signal during their transit through the oscillation zone.

When an auxin peak appears, fades, and then stabilizes again at the same position, the auxin peak marks a prebranch site (Moreno-Risueno et al. 2010), which is considered a predictor of where a new LR will form, and the amplitude of the auxin peak is correlated to a successful LR formation (Xuan et al. 2015).

The mechanism that determines LR positions remains unsolved. It has been proposed that auxin accumulation in certain pericycle cells within the differentiation zone triggers founder-cell specification and LR initiation, thereby establishing the spatial pattern of LR formation (Dubrovsky et al. 2008; Laskowski et al. 2008 ). Others hypothesize that there are patterning mechanism at the elongation zone, such as the priming step, where cells get primed at regular intervals (De Smet et al. 2007). In addition, the set of branching sites might be endogenously regulated by a “clock” with a stable, auxin-independent periodicity (Moreno-Risueno et al. 2010). There are experimental results that partially support the hypotheses, but how they fit together is still not clear.

Reporter genes driven by *DR5* and its variant *DR5rev* have been used to monitor auxin levels and auxin signaling. They have been used to study where and when LRs form. The reporters provide different readouts. *DR5::GUS* produces a blue enzymatic stain in fixed tissues, marking, for instance, the periodic priming and founder-cell specification (De Smet et al. 2007; Dubrovsky et al. 2008). *DR5::LUC* generates luminescence for live imaging of rhythmic auxin pulses and stable prebranch sites, but *DR5::LUC* has low tissue-level resolution and does not completely match *DR5::GUS* patterns. *DR5rev::3xVENUS-N7* produces nuclear fluorescence and detects signal changes near the transition zone. The loss of fluorescence in the lateral root cap (the outermost cell layer surrounding the meristem) predicts future branching points (Xuan et al. 2016). Because these reporters emphasize different tissues and time windows and resolution, they do not always display identical patterns, underscoring the value of integrated analyses.

Recently, De Gernier et al. (2025) reported in *Plant Physiology the use of* a dual-reporter to live image seedlings, allowing simultaneous tracking of *DR5rev::3xVENUS-N7*, which resolved cell positions, tissues, and divisions linked to LRP development, and *DR5::LUC*, which provided a whole-root view by capturing auxin responses at organ level.

The authors selected a protoxylem cell expresing *DR5rev::3xVENUS-N7* showing fluorescence, while adjacent cells did not show fluorescence, in the root tip. The selected cell was tracked over time and defined together with adjacent nonfluorescent pericycle cells as a group of primed cells (priming event). As primed cells entered the differentiation zone, the *DR5rev::3xVENUS-N7* signal disappeared from the protoxylem and appeared in the pericycle, consistent with founder-cell specification (Dubrovsky et al. 2008). Later, those pericycle cells underwent LR initiation. Simultaneously, *DR5::LUC* luminescence did not coincide with the earliest priming event. The *DR5::LUC* signal appeared as primed cells moved through the elongation zone, reached an auxin maximum near the differentiation zone, then decreased, sometimes preceding prebranch-site formation. Kymographs showed that some *DR5::LUC* signal peaks were followed by aborted prebranch sites characterized by weak or rapidly fading luminescence.

Independent priming events marked with *DR5rev::3xVENUS-N7* fluorescence and auxin peaks monitored with *DR5::LUC* luminescence were quantified to link them with LR outcomes. On average, priming occurred ∼238 µm from the quiescent center, while auxin peaks appeared further shootward at ∼830 µm. Auxin peaks spanned 2 to 4 protoxylem cells; then pericycle fluorescence (founder-cell specification) appeared ∼1.9 h later, prebranch sites formed ∼2.25 h after the auxin peak, and the first anticlinal division occurred ∼8.5 h after the peak. Compared with ongoing sites, aborted sites showed weaker and delayed *DR5::LUC* increases, although the timing of founder-cell specification was similar. The auxin peak amplitude and the ∼2.5-h interval between peaks did not differ between aborted and ongoing sites.

Additional analysis indicated that ongoing prebranch sites maintained *DR5::LUC* expression after LR initiation and supported LRP development, whereas aborted prebranch sites stopped after a single founder cell division. These aborted sites were more frequent in straighter primary root regions where vascular cells were longer, suggesting that local curvature and cell length influence success. Root curvature did not correlate with auxin signal strength of *DR5::LUC* or so-called auxin peak amplitude, and founder-cell orientation relative to the medium (agar or air tested) did not differ between ongoing or aborted prebranch sites. Thus, in wild type, auxin peak amplitude alone was insufficient to predict whether an LRP will develop.

Guided by the observation, and because ethylene is known to restrict root cell elongation enhancing auxin biosynthesis and transport (e.g. Bennett et al. 1996; Stepanova et al. 2008), the authors examined ethylene-related mutants. They focused on ethylene-overproducing *eto2* mutants (Vogel et al. 1998) and *pPIN2::gACS5eto2* lines (epidermal-cortical expression of *eto2* mutation). These lines had shorter, straighter primary roots and fewer total LRs than wild type, while LR density (per cm) was unchanged. *DR5::LUC* signal peaks occurred less frequently, with longer intervals, linking peak timing to elongation rate. Despite similar prebranch-site density in the higher-ethylene mutants, they showed more aborted prebranch sites; this suggests that ethylene may increase auxin response peak production, but the reduced curvature limits successful LRP development, balancing LR density overall.

This study revisits how DR5 reporters describe LR formation and proposes a unified framework. The authors align single-cell events with whole-root auxin dynamics: priming occurs first in the transition zone, the auxin-response peak appears later as primed cells reach the oscillation zone, and a stable DR5 signal at a prebranch site marks founder-cell specification and ongoing LR formation in the differentiation zone (Fig. 1). The study suggests comparing auxin-response peak rhythmicity by peak density per unit root length rather than by the time between peaks, which depends on elongation rate. It also highlights unresolved questions, including how signals move from protoxylem or lateral root cap to pericycle, how curvature promotes founder-cell initiation, how auxin interacts with ethylene across these steps, and whether the model extends beyond Arabidopsis. Finally, it offers a cell-by-zone map and a dual-reporter framework to guide experimental design while noting that spatial resolution, particularly for luminescence, could be further improved.

## Data Availability

No additional data were produced or assessed to support this research.
